# A DIY Low-Cost Wireless Wind Data Acquisition System Used to Study an Arid Coastal Foredune

**DOI:** 10.3390/s20041064

**Published:** 2020-02-15

**Authors:** Antonio C. Domínguez-Brito, Jorge Cabrera-Gámez, Manuel Viera-Pérez, Eduardo Rodríguez-Barrera, Luis Hernández-Calvento

**Affiliations:** 1 Instituto Universitario de Sistemas Inteligentes y Aplicaciones Numéricas en Ingeniería (SIANI), Universidad de Las Palmas de Gran Canaria, 35017 Gran Canaria, Spain; jorge.cabrera@ulpgc.es (J.C.-G.); eduardo.rodriguez@ulpgc.es (E.R.-B.); 2 Departamento de Informática y Sistemas, Universidad de Las Palmas de Gran Canaria, 35017 Gran Canaria, Spain; 3 Grupo de Geografía Física y Medio Ambiente. Instituto de Oceanografía y Cambio Global, IOCAG, Universidad de Las Palmas de Gran Canaria, Unidad Asociada ULPGC-CSIC, 35017 Gran Canaria, Spain; viera.manolo@gmail.com (M.V.-P.); luis.hernandez.calvento@ulpgc.es (L.H.-C.)

**Keywords:** DIY, low-cost sensors, wind sensors, environmental monitoring, arid foredune, tongue dunes, digital elevation model (DEM)

## Abstract

Environmental studies on coastal dune systems are faced with a considerable cost barrier due to the cost of the instrumentation and sensory equipment required for data collection. These systems play an important role in coastal areas as a protection against erosion and as providers of stability to coastal sedimentary deposits. The DIY (*Do-It-Yourself*) approach to data acquisition can reduce the cost of these environmental studies. In this paper, a low-cost DIY wireless wind data acquisition system is presented which reduces the cost barrier inherent to these types of studies. The system is deployed for the analysis of the foredune of Maspalomas, an arid dune field situated on the south coast of Gran Canaria (Canary Islands, Spain), for the specific purpose of studying the dynamics of a dune type (*tongue dunes*), which is typical of this environment. The results obtained can be of interest for the study of these coastal environments at both the local level, for the management of this particular dune field, and at the general level for other similar dune fields around the world.

## 1. Introduction

In recent decades, important environmental changes induced by human activities have been detected in coastal dune systems around the world, resulting in their degradation and, in some cases, their total destruction [1,2]. However, these dune systems play important roles in protecting coasts [3], providing stability to coastal sedimentary deposits. From the point of view of their conservation, it is essential to know the natural processes that take place in these systems, especially those related to the functioning of key landforms, as is the case of foredunes that provide different ecosystem services, including coastal protection against erosion and the maintenance of the sedimentary balance of entire dune fields [4].

Data acquisition in extensive natural environments, as is the case of foredune systems, can be an expensive process due to the generally high cost of commercial instruments and sensors with an adequate spatial and temporal resolution. Hence, the main objective of this study is to present and use a set of affordable, low-cost DIY (*Do-It-Yourself*) sensors and instruments for wind data acquisition (speed and direction) in a coastal arid foredune system, and compare it with other approaches described in Section 2. Section 3 will describe the DIY low-cost system we have designed and built for this purpose. Section 4 will detail the experimental operative field tests carried out so far with the system and discuss the results that were obtained. Finally, Section 5 will present the conclusions.

## 2. Related Work

The proliferation in recent years of low-cost electronic hardware prototyping platforms has promoted and fostered the application of DIY approaches in many areas of research [5]. The paradigmatic platforms which best represent this trend are the microcontroller-based Arduino boards [6,7], and the GNU/Linux-based Raspberry Pi boards [8]. Both platforms allow the use of standard open-access programming environments for software building and, in the case of Arduino, there is even an IDE (*Integrated Development Environment*), based on C/C++, specifically designed for newbie programmers and electronic tinkering beginners. These platforms can also be easily integrated with all kinds of hardware, from sensors and actuators to communication hardware interfaces including serial buses (CAN [9], I2C [10], SPI [11], etc.) and many types of wireless links (Bluetooth [12], WiFi [13], XBee [14], etc.). It is even possible to carry out the integration of different hardware in a modular way, as is the case with Arduino *shields*. In addition, both platforms are cheap and have an extensive user base ranging from expert gurus to starting beginners who share documentation, projects, hardware designs, applications and software libraries—all publicly available on an open-source hardware/software basis in user forums and websites. These platforms are easily accessible in terms of monetary cost and have the added benefit of expert support and user software licenses, as there is plenty of software and information openly and freely available and a wide active user community which willingly offers its support to any prospective user.

In a more general sense, the DIY approach, with its open hardware and software nature, has the obvious advantage of a lower monetary cost compared to more expensive commercial instrumentation. At the same time, however, although they are free to use and modify, open hardware/software tools and libraries come with no guarantee or user support, and so DIY researchers need to invest time and effort to acquire enough knowledge and know-how to ensure their correct operation and behavior. Likewise, such tools and libraries are usually supplied with licenses, such as GPL and LGPL, which can be seen as restrictive in terms of commercial use [15]. Even so, DIY approaches are being applied to an increasing number of research problems, especially in the development of low-cost instrumentation, as they are a cost effective way of developing early prototypes and “proof of concept” instrumentation in comparison with commercial alternatives. Cost, however, is not the only factor to be taken into account. An additional motivating factor is the relative ease of the learning curve for DIY tools and software compared to more professional tools, allowing non-specialist researchers to build a working prototype from scratch using tools that are not endowed with features present in more professional ones. An illustrative example is the well-known Arduino IDE that does not integrate JTAG’s debugging mechanisms [16], an industrial standard present in most professional embedded system development tools. Importantly, the DIY approach is potentially applicable in many fields. For instance, with respect to medical applications, Sweeney et al. [17] describe the development of a cost-effective alternative for measuring blood clotting time in patients. For museums, a low-cost surveillance and information system is presented in [18] which protects paintings from camera flashlights and visitors who get too close to the paintings. Another example, for oceanographic applications, is the development of a fluorometer built using low-cost electronics and used to estimate phytoplankton biomass [19].

Environmental monitoring is a field where the application of DIY approaches is particularly cost-effective, as it is usually necessary to deploy many sensor devices to adequately cover the area under study. In this context, the use of commercial devices is only affordable for research groups and institutions with substantial budgets, especially when many of them need to be deployed. There are several examples of recent DIY projects in this area of application. One is the Cave Pearl Data Logger [20], an Arduino-based data acquisition platform for subaquatic environments, which has already been successfully deployed to measure drip rates in subterranean cave systems and water flow in flooded caves. Each sensor unit, according to its authors, costs less than $50 per device and its design, firmware, and software is openly available to researchers, educators, students and ordinary users. Similarly, a wireless acoustic sensor network (WASN) is presented in [21], consisting of Raspberry Pi-based devices for measuring noise pollution in urban environments. Another example is the KdUINO DIY Buoy [22], an instrument used to quantify water transparency by estimating the diffuse attenuation coefficient using irradiance measures at different water depths. Its creators underline the fact that a KdUINO costs about $100, around 10 times less than an equivalent commercial instrument. In [23], another DIY platform for oceanographic applications is described, in this case a multi-purpose platform capable of taking different environmental measurements in coastal areas. To date, it has been used in two different setups, as a Lagrangian drifter for acquiring salinity and temperature data and as a moored platform for longer deployments. Its low cost, about $300, in comparison with commercial instruments, allows several of them to be built and deployed with a relatively low expense. Another recent application of the DIY approach is the work described in [24], which presents the design and development of a DIY sedimentary trap instrument, able to collect wind-transported sand in dune fields and send the corresponding data in real time using a wireless sensor network (WSN). In another work, Chan et al. [25] advocate the use of the DIY approach for building low-cost electronic sensors for environmental applications. The authors describe six case studies—a water depth probe, an air quality logger, a water quality logger, a multi-parameter weather station, a lake sediment trap design, and a logger for monitoring sand transport—and the lessons that were learnt. In general, the affordable cost of the DIY approach, together with the open nature of designs, software, and user support, makes such devices ideal for citizen science programs and projects [26,27,28].

Usually, wind data acquisition in dune systems is carried out using 2D and 3D ultrasonic anemometers [29,30,31,32]. Ultrasonic anemometers present important data acquisition features compared to the more classical cup-type anemometers; namely more precise measurements, higher sampling frequencies and three-dimensional wind data in the case of 3D devices, making them suitable for measuring wind turbulence properties [33]. In some works, ultrasonic anemometers are deployed in combination with cup anemometers [34,35], with the latter mainly used for wind speed sampling in the less turbulent locations of the study area, commonly mounting several of them at different levels on towers or masts situated on dune crests or windward. The cost of commercial meteorological instruments for wind data acquisition, particularly in the case of top-of-the-line instrumentation used in previously cited works, whether cup or ultrasonic type, constitutes an important cost barrier for studies in coastal areas dominated by dune fields, where the wind is a fundamental factor in the dynamics of the system. Furthermore, in addition to the cost of the wind instruments themselves, the need for other essential equipment has to be taken into account, including data loggers and communication links, whether wired—normally serial links—or wireless -like radio communications, Bluetooth, or WiFi networking devices. These devices are usually provided as complementary equipment of the wind instruments themselves, and so the cost of the whole data acquisition system increases. The DIY approach can contribute to lowering the instrumentation cost barrier in such studies. With all the above in mind, in this paper, we present a wireless low-cost DIY approach for wind data acquisition which has been specifically applied to the study of the foredune system of an arid coastal dune field located in Maspalomas (Gran Canaria, Canary Islands, Spain).

## 3. Material and Methods

In most sandy coastlines of temperate and tropical regions of the world, foredunes are continuous landforms aligned with the coastline. Plant species commonly found in these systems include the graminaceous *Ammophila arenaria* and *Elymus farctus*. When the vegetation density is low, a continuous linear dune field does not appear but rather a set of *nebkhas* [36,37], a type of sand dune that forms around vegetation. These features are usually related to a positive balance of sand and moderate wind energy, with scarce or ineffective vegetation cover [36]. When formed in temperate zones, these nebkhas have been defined as an erosive phase of the dune field that is typical of these environments [38]. However, they can also be found in arid regions, where continuous foredunes do not build up [38,39,40]. Such is the case in the Canary Islands, where the dry climate conditions present at coastal level only allow the formation of nebkhas, mainly associated with the nanerophytic bush *Traganum moquinii* [41,42,43,44]. The habitat of this species is basically the coastal dunes, from the backshore to around 200 meters inside the dune field. Its worldwide distribution is constrained to the NW coast of Africa, from Morocco to Mauritania, and it is also found in the Canary Islands and Cape Verde archipelagos [43].

The role played by *Traganum moquinii* in the formation of foredunes in arid coastal dune systems of these regions was reported in a study of the relationship between specimens of this plant found in Playa del Inglés (Gran Canaria, Canary Islands, Spain) and the wind dynamics, in which it was observed that the influence of these plants extends 20 m leeward of their placement [45], generating a parabolic dune shape, or *tongue dune* [46]. The identification of tongue dunes within this scheme was a new finding because parabolic dunes are normally associated with wet aeolian sedimentary systems, where the parabola shape is due to the generation of a frontal lobe in a blowout. When these forms show up in the foredune, it is usually due to the rupture of the longitudinal continuity of the foredunes in tropical and temperate regions. In our case, however, these dunes are formed spontaneously and hence are characteristic landforms of arid region foredunes. They are indicative of the existence of a notable sediment input and, together with the nebkha dunes, form protective barriers against marine erosion. In addition, they give these transgressive systems a particularity: inland that is leeward of the foredune zone, the dunes evolve into free dunes, acquiring the morphology of barchan dunes. When plant specimens are uprooted in the foredune, the disappearance of these dunes occurs and, consequently, instead of the aforementioned barchan dunes, sheets of sand are formed inside the system which are transported at higher speeds [47]. The characterization of tongue dunes is therefore needed in these arid environments, especially given the role they can play in the face of a climate change-driven rise in sea level.

Considering these premises, this paper addresses the interaction of wind with specimens of *Traganum moquinii* in these spaces with a view to explaining the formation of tongue dunes. In order to achieve this goal, a DIY approach has been applied to wind data acquisition for the purpose of validating a theoretical model of this interaction using topography and wind data. As the foredune of arid dune fields has been little studied, the results of this study can be useful for the management of the Maspalomas dune field, as well as other coastal dune fields around the world.

### 3.1. Wind Data Acquisition System

Environmental monitoring of an area, in this case for the study of a dune field, requires the deployment and spatial distribution of a sufficient number of wind sensors over the study area to adequately sample aeolian conditions over the dunes at different heights. In other words, a large number of wind sensing devices is generally required. As the use of standard commercial scientific-grade sensors was not viable under our budget constraints, we decided to explore the construction of our own wind data acquisition system following a low-cost DIY approach. The system consists of a base station and several independent wireless wind sensing devices. Eleven of these devices were developed and built for the work described in this paper.

#### 3.1.1. Design Principles

The design of the wireless system was driven mainly by the following initial premises and requirements, determined principally by the task at hand:

**Cost** The funding available for developing the system was strictly limited. This was a requirement which affected all aspects of the design process. As our research interests were focused on strong or very strong wind conditions under which sand transportation occurs (more than 5 m/s), our field experiments did not require wind sensors of extreme sensitivity. However, a large number of sensors were needed, and we therefore decided to resort to a DIY approach and the use of low-cost electronics and instruments.
**Flexible parametrization system** The final system should be flexible enough to parameterize the data structure, as for example the data sampling periods, log files, etc.
**Autonomy** The system, and specifically each independent wireless sensor device, should have sufficient autonomy, in terms of energy consumption, to allow continuous on-field session measurements of at least 10 h.
**Temperature concerns** The system needed to endure high temperatures during the day. A temperature control system for each wireless sensor device was therefore an important requirement during deployment, and especially when a complete on-field data acquisition session was being undertaken.
**Easy deployment and use** Given the hard logistical conditions of the field where the system was to be used (especially due to the mobility of the substratum), the deployment of the data acquisition system needed to be as easy as possible. In addition, the system had to be user friendly given the multidisciplinary profiles of the people involved in the on-field measurement sessions.
**Wireless communications** There are numerous advantages to the use of wireless communication links between the elements of the system as opposed to wired connections, especially in terms of transportation and on-field deployment. Given that the wind sensing devices basically only need to periodically transmit wind speed and direction data, which require only a few bytes, and some complementary information such as timestamps, high data rate communication was not a strict requirement. As the aim was for the wind sensing devices to have a high degree of autonomy, a further requirement was to choose a wireless technology which consumed as little power as possible.
**Reach** The area to be covered in a typical data-acquisition task of this type is about 50 × 50 square meters. This was considered sufficient to study a whole foredune in the study area, as will be seen. Hence, the reach of the wireless communication system should at least cover these dimensions.
**Reliability** The experiments would be carried out under demanding conditions of intense sand/dust transportation, high temperatures, and strong winds. The system therefore had to be able to alert the user in case any of the wind sensing stations stopped transmitting. In fact, this proved to be an extremely useful feature which ensured the quality of the data that was obtained. Moreover, it is important that the data acquired during an experimental session is observable on-site as it is collected, in addition to being saved in a secondary memory device for later analysis. The detection and on-site fixing of errors is an important advantage which is not possible in systems in which access to collected data are only possible once the experiment has concluded.

#### 3.1.2. The Data Acquisition System

**Figure 1:**
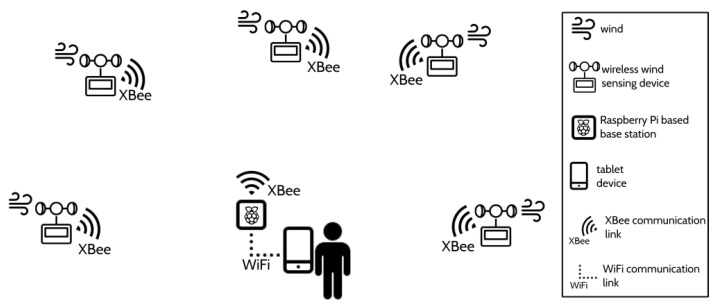
The wind data-acquisition system (icons from icons8.com).

Figure 1 depicts a graphical view of the data acquisition system that we developed. For illustrative purposes, only five wireless wind sensing devices are depicted although, as previously commented, eleven identical devices were developed and built. As can be seen, the data acquisition system consists of two kinds of nodes or elements: a *base station* and several *wireless wind sensing devices*. Both types are briefly explained below.

**Base station** The base station is a personal computer or similar device running GNU/Linux and equipped with an XBee communication link, which is used for system synchronization and monitoring during a data acquisition session. Usually, we deploy the base station running on a GNU/Linux Raspberry Pi based embedded computer device exporting its graphical environment via VNC (Virtual Networking Computing) [48] on a mobile tablet device. In this case, the embedded computer acts as a WiFi access point for the tablet. In contrast to the use of a laptop, this facilitates the on-field use of the base station given the harsh conditions during a data acquisition session of intense sand and dust transportation combined with strong winds. In this manner, it is possible to protect the embedded computer from sand, dust, and wind by keeping it in a backpack, for instance, and the tablet by using a convenient weather-proof cover.
**Wireless wind sensing devices** Each wireless wind sensing device is an independent, small Arduino-based embedded system for wind speed and direction data acquisition, which communicates periodically with the base station via XBee during a data acquisition session.

**Figure 2:**
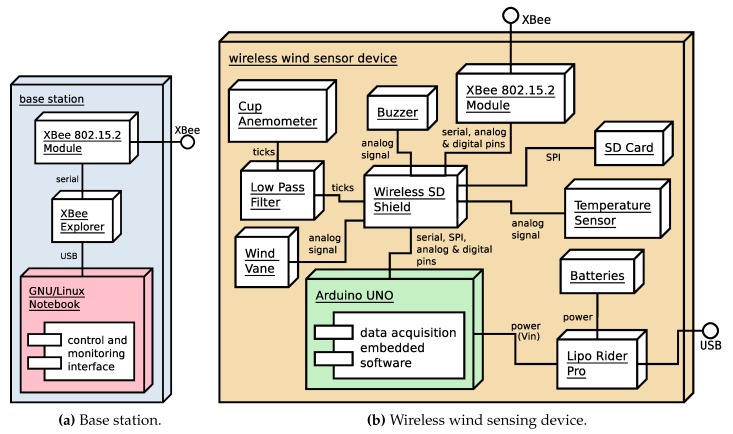
Wind data acquisition system. Deployment diagrams.

**Figure 3:**
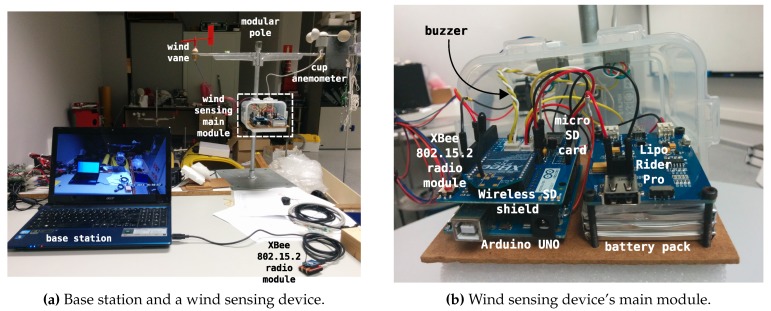
Base station and wind sensing device.

Figure 2a depicts the deployment diagram of the base station. From a hardware point of view, the base station is a computer running GNU/Linux with a USB adapter [49] to connect a XBee module for radio communications with the wind sensing devices (the notebook shown in Figure 3a). As explained previously, a convenient way to deploy the base station in a real data acquisition session environment is to use a Raspberry Pi-based embedded computer in combination with a tablet device to access its graphical interface via VNC (for purposes of clarity, this is not shown in the deployment diagram depicted in the figure). The XBee modules used at the base station and also at the wind sensing devices are XBee PRO S1 802.15.4 60 mW [50] modules with a line-of-sight reach of about 1.6 km. Bear in mind that, although initially the main requirement for the reach of the system was to cover an area of 50 × 50 square meters, XBee PRO S1 802.15.4 communication modules provide a longer reach, which allows the system to be deployed in wider areas. This is in contrast to other short-range wireless technologies, like Bluetooth, which despite providing higher data rates, have less reach and a higher power consumption [12,51,52,53].

**Figure 4:**
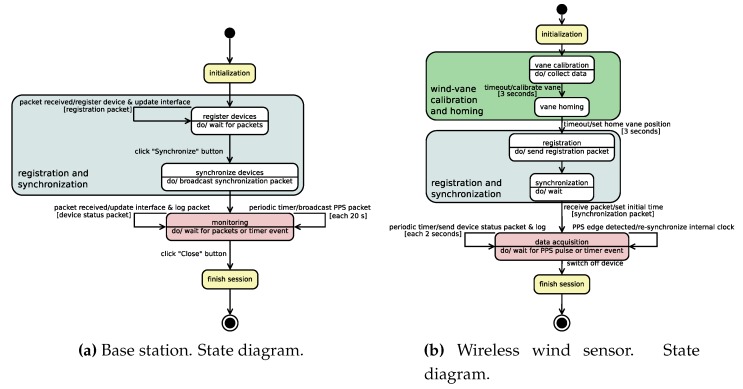
Wind data acquisition system—state diagrams.

The software developed for the base station constitutes a visual interface to control and monitor the wireless wind sensing devices during an on-field data-acquisition session. This software has been developed in C++ under GNU/Linux, using GTK [54], as graphical library, and *libxbee3* [55], a library to interact with the communication XBee module through the XBee USB adapter [49]. The functionality of the base station control and monitoring interface is outlined in the state diagram shown in Figure 4a, which represents its flow of execution during a data acquisition session.

The deployment diagram of each of the wireless wind sensing devices is shown in Figure 2b. One of the devices can be seen in Figure 3a, and is shown in greater detail in Figure 3b, where it is mounted on one of the modular poles used for deployment. These devices constitute the data acquisition elements of the system. Each is an embedded system built around an Arduino UNO microcontroller prototyping platform [56], where we integrated the hardware which appears in the diagram of Figure 2b. Specifically:

- a Wireless SD Shield [57] which allows the connection of an XBee radio communication module (the same model as used in the base station), an XBee PRO S1 802.15.4 60 mW [50] module, and a micro-SD card as secondary storage medium for data logging;
- a wind sensor for measuring wind speed and direction comprising a cup anemometer (weather station WS-2080 anemometer [58], accuracy: ±3.54 km/h, maximum measured speed: 180.25 km/h, resolution: 0.161 km/h) and a wind vane (based on a US Digital MA3 angular sensor [59])—a DIY design already used in robotic sailing navigation [60];
- an RC low pass filter to smooth out the cup anemometer signal;
- a buzzer to signal the wind vane calibration and homing processes when starting, or to indicate any device error condition;
- a temperature sensor;
- and a battery power supply system formed by a Lipo Rider Pro supply/charging board [61] and a 6000 mAh 3.7 v. battery pack, which provides a continuous autonomy of about 30 h.

With a view to obtaining as precise a location as possible of the wind sensing devices, once deployed on-field, we used a topographic total station to determine the geographical position of each device. Thus, the integration of a positioning sensor (a GPS receiver) in each device—which additionally would have provided a less precise positional accuracy—was not required. No real-time clock (RTC) was integrated either, as real time is usually obtained from the GPS receiver, and it is possible to avoid clock drifting between different sensors using the PPS (Pulses Per Second) signal available in GPS receivers [62]. In the absence of a GPS receiver, and in order to have a real-time reference in each wind station, we implemented an initial synchronization procedure between the base station and the wind sensing devices at the beginning of each data acquisition session. Analogously, to avoid clock drifting, which can be quite significant in Arduino UNO based devices [63], we implemented a “PPS signal” through one of the output digital pins of the XBee PRO S1 801.15.4 modules [50]. Subsequently, this signal was set/unset from the base station using a broadcast packet, keeping all sensor devices synchronized with the base station clock. In a scenario in which the configuration of each sensor with a GPS receiver is required, there would be no need for this initial synchronization procedure with the base station. Moreover, we also decided not to integrate a compass in each sensor device, as it was a simple procedure to set a reference for the orientation of each sensor’s wind vane using an external compass at the beginning of each measurement session, as will be explained later.

As to the power autonomy of each wind sensing device, we experimentally determined an autonomy of slightly more than 30 h with fully charged batteries. This is well above the 8–10 h maximum duration of the experimental sessions with the wind data acquisition system. In any case, if necessary, the Lipo Rider Pro supply/charging board, integrated in each device, allows the batteries to be charged with a solar panel in the range of 4.8–6.5 v. and 400–600 mA.

The embedded data acquisition software we developed for the wireless wind sensors runs on the Atmel ATmega328P microcontroller integrated on the Arduino UNO board. It was developed in C++ under GNU/Linux using the Arduino IDE [6], Arduino’s standard libraries and the *xbee-arduino* library [64]. The flow of execution of each of these wind sensors in a measurement session is depicted in the state diagram of Figure 4b.

During a measurement session, the data acquisition system has the topology shown in Figure 1. As already mentioned, communications between the nodes of the system are carried out using XBee radio links, through which data packets with specific formats are sent. Following a star topology, the wireless wind sensing devices do not communicate with each other but only with the base station on a point-to-point (P2P) basis, as can be seen in Figure 1.

The diagrams in Figure 4a,b clarify the different states of the system during a data acquisition session. To start a measurement session, the control and monitoring interface must first be initiated in the base station. The interface, once initialized, waits for the wireless wind sensing devices to be registered (state *register devices*, Figure 4a).

Next, each of the wind sensors we want to deploy for the session needs to be switched on. For each of them, initially, a wind vane calibration procedure needs to be followed (state *vane calibration*, Figure 4b). The procedure consists of spinning the wind vane at least 360 degrees in a time window of three seconds. This time window is signaled emitting a specific beep pattern from the buzzer at the beginning and end of the procedure. Following this, a reference direction for the wind vane needs to be established with an external compass (state *vane homing*, Figure 4b). A time window, again indicated by a buzzer emitting a beep pattern at the beginning and end of a 3-second time window, is provided to set the home position of the vane. To set this reference, the wind vane needs to be maintained during this time window indicating north as given by the external compass. Once the wind vane has been calibrated and *homed*, the device sends a registration packet to the base station (state *registration*, Figure 4b), and then passes to an idle state waiting for a clock synchronization packet from the base station (state *synchronization*, Figure 4b).

**Figure 5:**
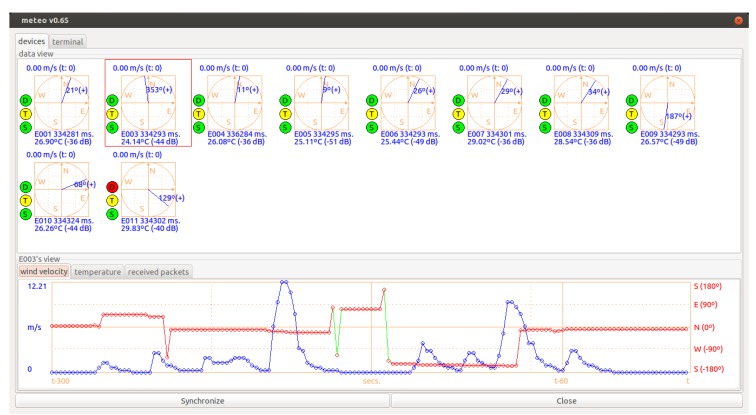
Base station. Control and monitoring interface. On the *data view*, each registered sensing device appears showing graphically the wind data the base station is receiving from it. By selecting a device in this view, we can see the last 5 min (300 s) of accumulated data below that specific device. The *data view* also provides for each device three “virtual” LED status indicators (coloured circles), namely: “S” for signal/communication status, “T” for temperature status and “D” for data reception status.

At the base station, for each registration packet which is received from each wind sensing device, the device is registered and appears on the visual control and monitoring interface (state *register devices*, Figure 4a). That is, as each device is deployed, installed and switched on, it appears on the base station interface. Once all the deployed devices have been registered (Figure 5 shows a snapshot of the interface with several devices already registered), we can make the system start acquiring data by clicking on the synchronization button of the interface. By doing so, the base station broadcasts a synchronization packet (state *synchronize devices*, Figure 4a), and then passes to a state of monitoring and control of the registered wind sensing devices while they acquire data (state *monitoring*, Figure 4a). The wind sensor devices, once they receive the synchronization packet broadcast by the base station, synchronize their internal time with the packet and start measuring (state *data acquisition*, Figure 4b).

During the data acquisition state (state *data acquisition*, Figure 4b), each wind sensing device samples the wind speed and direction for a specific period (2 s in the diagram). The sampled data are stored on each device’s on-board micro-SD secondary memory. In addition to the raw sampled data, median filters of 3 and 7 samples for wind speed and direction angle, respectively, are applied to the measurements. These filtered data are also stored together with other device status information (temperature, anemometer clicks, etc.). All logged information is time-stamped. Furthermore, a status packet is sent to the base station, which contains part of the information which has been stored in secondary memory (time-stamp, filtered wind data and temperature). During the same state, the device’s internal clock is re-synchronized each time a PPS edge change is detected through one of the XBee module’s digital output pins. This PPS edge-changing event is also stored in secondary memory in the device’s log. Finally, each device keeps measuring in this way until it is switched off by the user (state *finish session*, Figure 4b).

As mentioned, once the wind sensing devices are synchronized, the base station enters a system monitoring mode (state *monitoring*, Figure 4a). In this state, each time a status packet is received from a wind device, the interface is updated accordingly. In addition, the packet is stored in secondary memory in the base station. Throughout this same state, the base station keeps periodically broadcasting “PPS packets” to the wind sensing devices. These packets, once received on the devices’ XBee modules, generate edge changes on each module’s designated output digital pin to have the sensor devices’ internal clocks re-synchronized. The period of emission of these packets was set to 20 s, as Arduino UNO’s main clock is a 16 MHz ceramic resonator with a frequency tolerance of ±0.5% [65] that is an accuracy of 5000 ppm (Parts Per Million). A 20-s period allows a synchronized time to be maintained in the sensing devices with a maximum error of 100 milliseconds [63]. The base station keeps running in this monitoring state until the user explicitly finishes its execution (state *finish session*, Figure 4a), terminating in this way the data acquisition session.

To simplify the state diagrams of Figure 4a,b, we have not included error-related states. Fault situations may arise with the wind sensing devices, mainly at the initialization phase when the hardware is being checked and when there is a person installing the device and performing the wind vane calibration and homing initialization procedures. In the event of an error, a wind sensor signals the situation by emitting an SOS Morse [66] beep pattern using the buzzer. The monitoring software in the base station is able to detect various undesirable circumstances which could ruin a data acquisition session. This is a valuable resource, especially when the session is carried out in complicated real scenarios in terms of logistics for sensor deployment, and there is no easy physical access to them once they have been installed and successfully started. The inclusion of the possible diagnosis by the base station of a large number of errors ensures that, once the devices start acquiring wind data, they will be able to complete a whole data acquisition session without errors. A list of the undesirable error situations that can be detected using the monitoring interface which is executed at the base station is shown below:

- If a specific deployed wind sensor has been started but the base station has not received any registration packet from it. In this case, there is probably no good line of sight with the device, and so the solution is to move the base station or the device to another location.
- If a device sends data which does not change over time. This is a typical error and usually means that there is a connection problem with the anemometer or the wind vane. This is indicated on the interface by a virtual LED for each device (“D” status indicator in Figure 5).
- If the temperature is too high for a particular device. As this could result in device failure, the interface shows the temperature of each device, and a virtual LED (“T” status indicator in Figure 5) indicates the temperature level and turns red when is too high (greater than 70 ${}^{{}^{\circ}}$C).
- If status packets have not been received from a specific device for a given time duration, the situation is indicated through a virtual LED (“S” status indicator in Figure 5). This circumstance might be temporary, but if it persists the device could be suffering from a communication problem or be unavailable for use due to other causes. Note that communication problems can cause some PPS packets, broadcasted from the base station, to be lost, resulting in a device’s internal clock accumulating too much drift if the problems persist for an excessive time. In this case, it is possible, through offline data processing, to re-synchronize the data acquired by the malfunctioning device, combining the data logs stored by all the devices deployed in the experimental session.

### 3.2. Experimental Validation of Wind Sensing Device Measurements

With the aim of validating the measurements obtained with the wind sensing devices which comprise our DIY data acquisition system, we undertook an experimental comparison in real conditions with scientific-grade wind sensors, namely the Thies Clima anemometer model 4.3159.00.140 [67] and wind vane model 4.3129.60.140 [68]. These sensors were kindly provided by the Spanish meteorological governmental agency AEMET (*Agencia Estatal de METeorología* [69]), which commonly uses them for verification and validation of meteorological wind instrumentation in airports, ports and other geographical locations. AEMET personnel helped us to carry out this experimental validation with their sensors.

**Figure 6:**
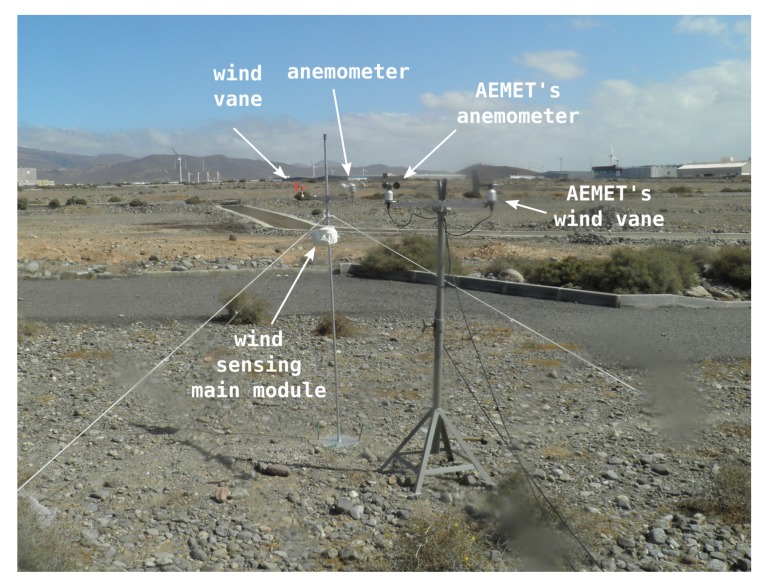
Experimental site during validation experiments.

**Figure 7:**
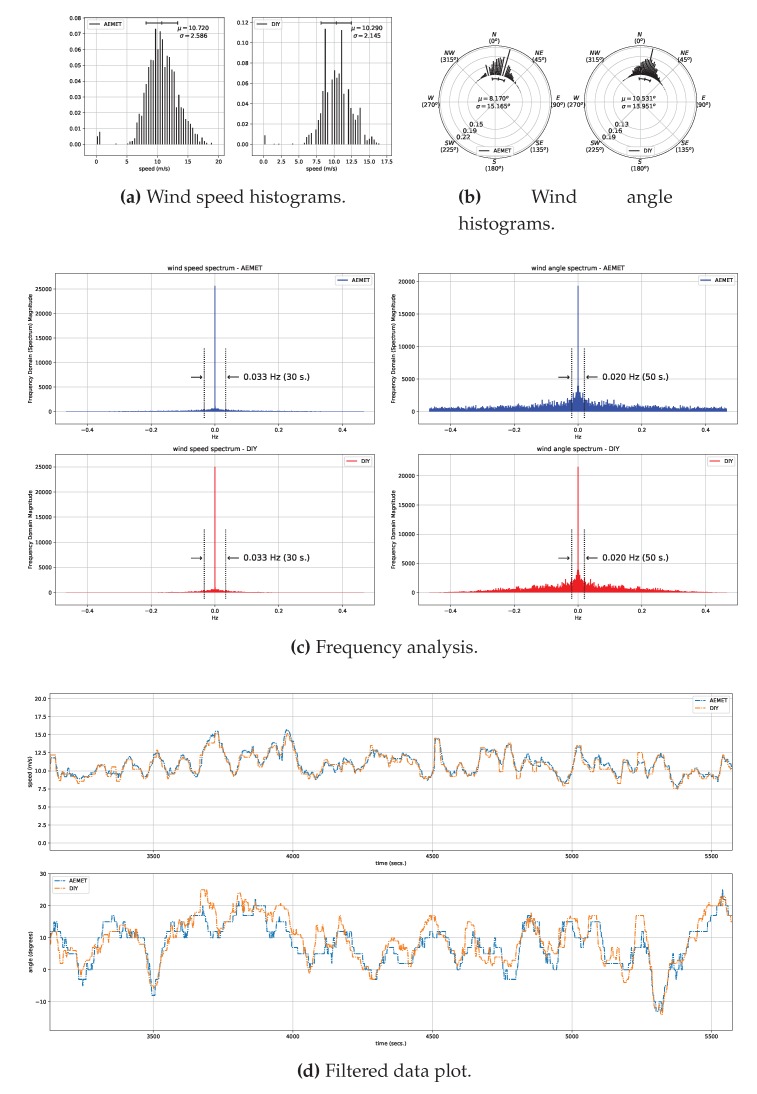
Data validation. (**a**,**b**) show respectively the normalized histograms for raw wind speed and direction angle data acquired by each sensor. Each histogram line represents the fraction of measurements in a discrete interval or bin of speeds and angles, respectively. Histogram bins are evenly spaced and have the same width; (**c**) shows the frequency analysis of collected raw wind speed and direction angle data, and the low-pass filter threshold applied; (**d**) shows a plot of the filtered data. In all figures, *AEMET* refers to AEMET’s sensors, and *DIY* to one of the wind sensing devices which make up our DIY data acquisition system.

For this validation, we conducted a data acquisition experimental session in Arinaga, a windy zone located south of Gran Canaria’s airport on the east coast of the island. Figure 6 shows the experimental setup that we deployed on site. Wind data was acquired using AEMET’s wind sensors and one of the wind sensing devices which make up our DIY data acquisition system. The wind direction in the area is mainly from the northeast. In order to reduce as much as possible inter-sensor effects, the arms, at whose ends each pair of sensors (anemometer and wind vane) was positioned, were aligned perpendicularly to this main wind direction, as shown in the figure. Figure 7a,b show the histograms of raw data collected during the experimental session which lasted approximately 90 minutes. Data were collected at 1 Hz for both types of sensors.

In order to make a comparison between the wind sensing devices of our data acquisition system and those of AEMET, we applied a low-pass filter to cancel out any possible noise present in the two sets of data, wind speed and direction angle, respectively, for both types of sensors. The filter applied was a centered median filter, and, with a view to determining the most suitable filter length, we performed a frequency domain analysis with application of the discrete Fourier transform to both data sets. Figure 7c shows the results of this analysis, with the 30- and 50-s thresholds that were chosen for the length of the median filters that we applied to the wind speed and direction angle data, respectively. As can be seen, the wind direction data are noisier, and so we chose a longer filter length. For visual comparison, Figure 7d shows the data acquired during the validation experimental session by both AEMET’s sensors and our own, once processed and after applying the aforementioned filters to both sets of data, wind speed and direction angle, respectively. Finally, we obtained the results shown in Table 1 for the error of the measurements. We considered these results to be a good fit for the wind speed and direction angle, bearing in mind that the small differences may well have been due, at least to a certain extent, to the sensors not being mechanically identical and not being situated at exactly the same position, especially in the case of the wind direction angle data as AEMET’s wind vane and ours were situated approximately two meters apart, as can be seen in Figure 6.

## 4. Results and Discussion

The development of this DIY data acquisition system has been motivated and driven by real scientific demands. This section describes the results obtained with it in the context of a specific environmental research study.

### 4.1. Study Area and Experimental Methodology

**Figure 8:**
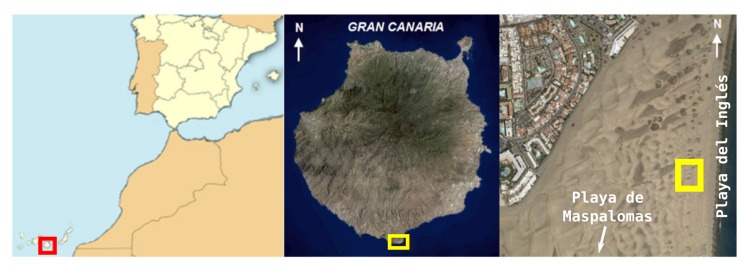
Study area and experimental site location (right-hand image, in yellow).

The Maspalomas transgressive [70] dune field is located in the southern vertex of Gran Canaria (Canary Islands, Spain) (Figure 8), on an alluvial plain composed of marine and terrestrial sedimentary deposits whose origin has been dated to the Quaternary [71,72]. The Maspalomas dune field is covered by moderately well classified and symmetric fine sands, although with some tendency to negative asymmetries. The granulometric distribution is clearly unimodal, which confirms the aeolian character of these materials [73].

Sands enter the system from the east, in Playa del Inglés (El Inglés beach), are transported in a NE-SW direction as free dunes at a mean rate of 7.93 m/year [73], and finally return to the sea via Playa de Maspalomas (Maspalomas beach) to the south of the dune field. The high transport rates are possible due to the climatic specificities of this system. In the dune field, a large variety of free dunes can be found, with barchan dunes and barchanoid and transversal ridges the most noteworthy [72]. Playa del Inglés is a wide beach with a stable smooth slope from a sedimentary point of view [74]. In the backshore, there is a monospecific shrub community of nanerophitic *Traganum moquinii*. This species is responsible for the formation of nebkhas [44,75], typical of the foredunes of the Canary and Cape Verde archipelagos as well as northwest Africa, and also for the formation of parabolic-shaped dunes [46].

**Figure 9:**
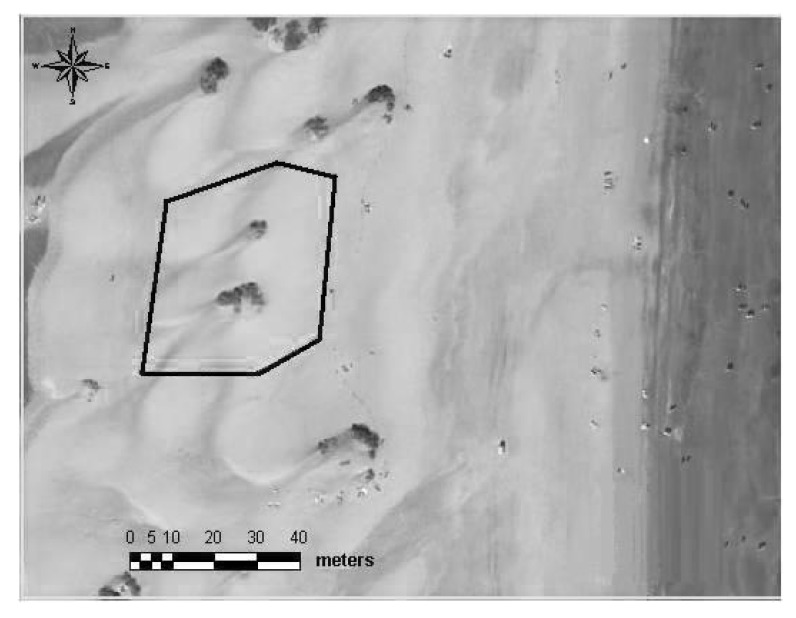
General view of the experimental plot. (Source: GRAFCAN S.A.).

For this experimental study, an area was selected in the mid-zone of the dune system, highlighted in the right-hand side image of Figure 8, and shown in more detail in Figure 9. Two experimental sessions were carried out the same day. Firstly, a topographical survey was developed using a total station. Then, wind data (speed and direction) were collected using the DIY data acquisition system described in the previous section, namely the eleven wireless wind sensing devices built and developed specifically for the task. The selected plot covered 1782.8 square meters and was chosen for the following reasons. First, given what has happened in the central sector, the northern and southern dunes have been severely affected by tourism development [43,47]. Second, the plot is located sufficiently far from the tourist areas and the zones used for sunbeds and parasols to minimize the risk of interaction with anthropic activities during the experiments. Third, the main interest of this experimental analysis was to analyse the formation of tongue dunes in this natural environment, a type of dune formed between two nebkhas identified in a previous study [44,46] in the same area.

**Figure 10:**
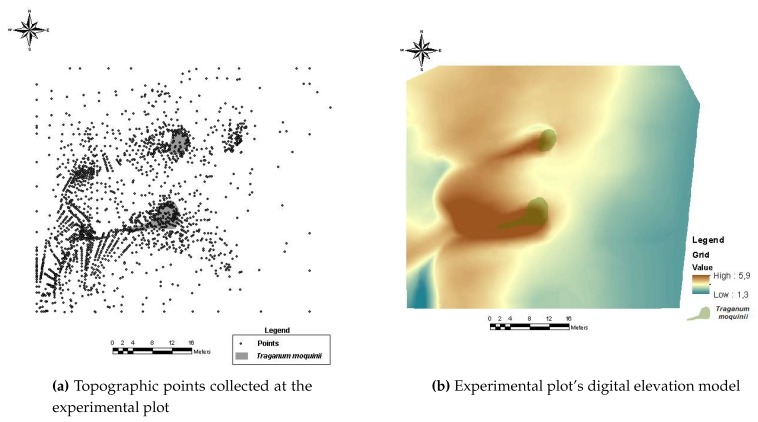
Experimental plot topographic survey and DEM.

A Leica TS06 total station model [76] was used to carry out the topographical survey. Its integrated laser allows the topographic points to be acquired without the need to step on the terrain, thereby avoiding any modification of the landforms. The collection of the points was conducted using the radiation method from a fixed-located station and different free stations. The fixed-located station had the following Universal Transverse Mercator (UTM) coordinates: X=443895,586, Y=3069206,613 UTM zone 28N and International Terrestrial Reference System (ITRS) 93. *Z* was 1.420 m, based on the Mean Sea Level (MSL) of Puerto de La Luz y de Las Palmas, taken using a GPS device. The topographical method applied was an inverse intersection from distances. A larger number of points were collected in areas with higher geomorphological complexity to ensure a more accurate model. A total of 2100 points were acquired for the elaboration of a Digital Elevation Model (DEM) in vector format (TIN) and later in raster format (Figure 10).

#### Data Acquisition Sessions

The data acquisition sessions for this experimental study were carried out on 8 November 2014. Two experimental sessions were completed. In both cases, a wind sensing device as control was located windward from the hillock dune at a distance sufficiently large as to not generate significant wind interference with the landforms.

**Figure 11:**
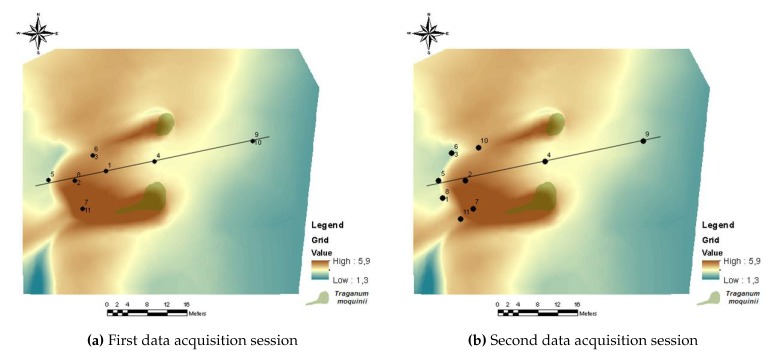
Wind sensor locations in both data acquisition sessions. The line shown in both figures is the longitudinal axis of the dune.

**Figure 12:**
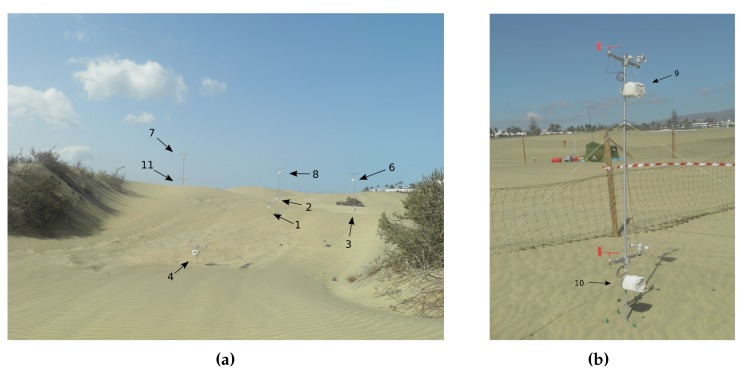
Photographs of the deployed devices at the experimental site during the first data acquisition session. Following the geographical layout depicted in Figure 11a, the left-hand image, (**a**), shows on either side the *Traganum moquinii* specimens at the two extremes of the dune, as well as eight of the eleven devices which were deployed, namely devices 1, 2, 3, 4, 6, 7, 8, and 11. Device 5 is occluded behind the dune and does not appear in the image. In the image on the right-hand side, (**b**), the last two devices, 9 and 10, are shown.

**Figure 13:**
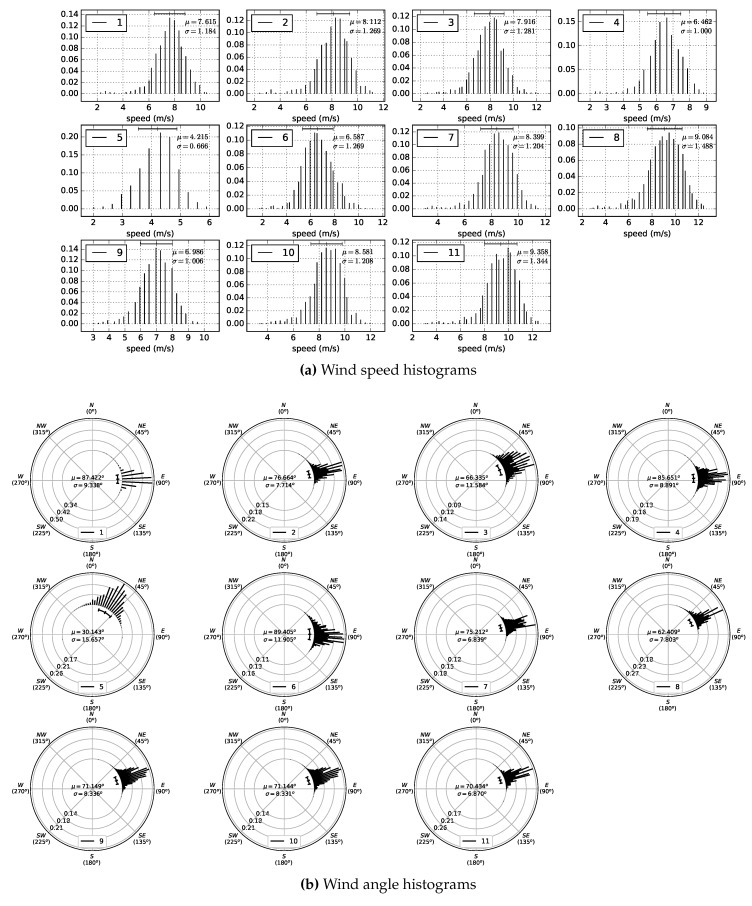
First data acquisition session. Time period: 10:30 a.m.–12:17 p.m. Eleven sensors were used in this experimental session. (**a**) and (**b**) show respectively the normalized histograms of wind speed and direction acquired by each sensor during the experiment. Each histogram line represents the fraction of measurements in a discrete interval or bin of speeds and angles, respectively. Bins are evenly spaced and have the same width.

**Figure 14:**
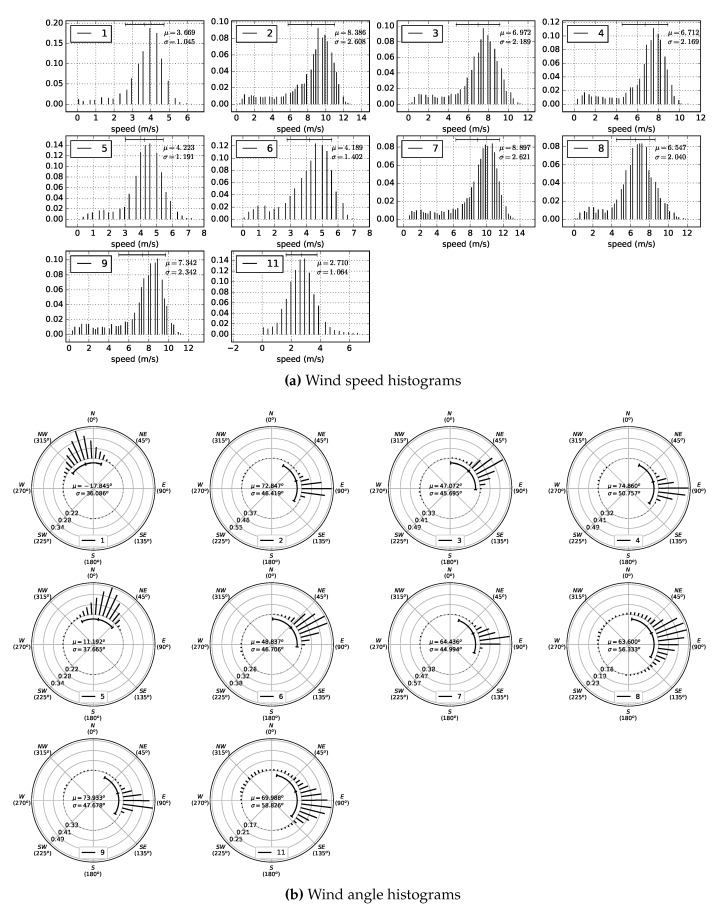
Second data acquisition session. Time period: 1:00 p.m.–4:38 p.m. Ten sensors were used in this experimental session (eleven sensors were used, but sensor 10 malfunctioned during this session and so has been omitted). (**a**,**b**) show respectively the histograms of wind speed and direction acquired by each sensor during the experiment. Each histogram line represents the fraction of measurements in a discrete interval or bin of speeds and angles respectively. Bins are evenly spaced and have the same width.

The first data acquisition session, corresponding to the first experiment, was performed from 10:30 a.m. to 12:17 a.m. Four poles were used, some with two different wind sensors at different heights, to obtain information about the wind profile. The data acquisition session for the second experiment was carried out from 1:00 p.m. to 4:38 p.m. In this session, the emphasis was on analysing the wind at points where, a priori, there were inflections in wind speed and/or direction. Table 2 shows the UTM coordinates, the height with respect to the MSL, and the height relative to the terrain of each of the wind sensing devices that were deployed. Figure 11 depicts their locations, using as reference the DEM generated previously. Figure 12a,b show two photographs of the wind sensing devices deployed on-field in operation for the first data acquisition session. Figure 13 and Figure 14 summarize the data acquired with all the sensors deployed in both experimental sessions.

Finally, it should be noted that, given that temperature was an initial design concern for the data acquisition system, as commented previously, temperatures were also collected during both experimental sessions. Once we analysed, during offline processing, the distribution of temperatures recorded for each wind sensing device, the highest temperatures measured were well below 50 ${}^{{}^{\circ}}$C, so no device was in danger of overheating.

### 4.2. Wind Field Model

A mass consistent wind field model [77] was applied to perform a simulation using data obtained from the wireless sensor system. Wind data are used to construct an initial wind field $u_{0}$ using a horizontal interpolation and a vertical extrapolation. The horizontal interpolation is formulated as a function of the inverse of the squared distance to the measurement stations, and the inverse of the height differences. For the vertical extrapolation, a log-linear wind profile is considered which takes into account the horizontal interpolation and the effect of roughness on the wind speed. In this work, two different values of roughness were considered, one corresponding to the sand and the other to the vegetation.

**Figure 15:**
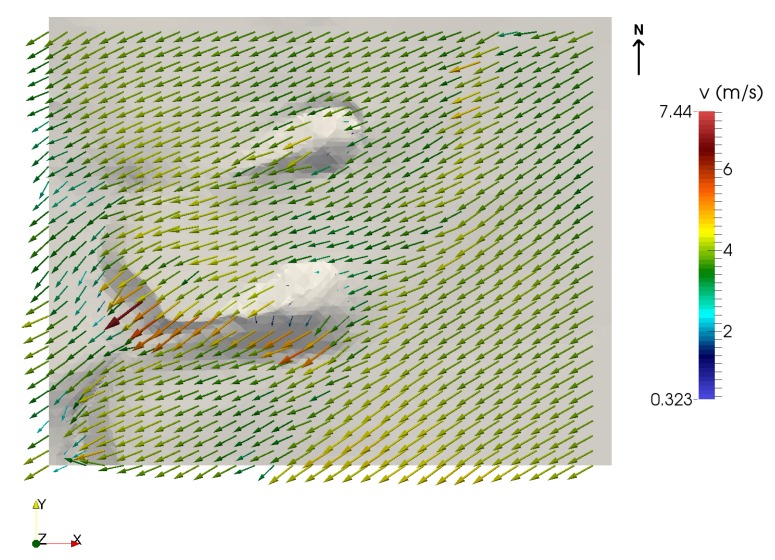
A view of the resulting wind field near the terrain.

A least-squares problem is formulated to find a wind field u, such that it is adjusted as much as possible to the interpolated wind field $u_{0}$, verifying the continuity equation in the domain and the impermeability condition on the terrain. This problem is solved by applying the Lagrange multiplier technique, leading to an elliptic partial differential equation which is solved with the Finite Element Method (FEM). In this work, to apply the FEM, an adaptive tetrahedral mesh of the domain was generated using the Meccano method [78,79]. This mesh has 124,898 elements and 24,545 nodes, with the smallest elements concentrated where more precision is needed, i.e., near the terrain. After calibrating the model with a genetic algorithm [77], computation of the wind field was performed. As a result, it was possible to calculate, through this model, the wind field near the terrain using the collected data in any given instant of time of the experimental set, as illustrated in Figure 15. That is, data obtained using the DIY data acquisition system and the wind model allow for the construction of wind fields in the domain, which can be used to study the wind dynamics in the area and their influence on the dunes.

### 4.3. Experimental Results

**Figure 16:**
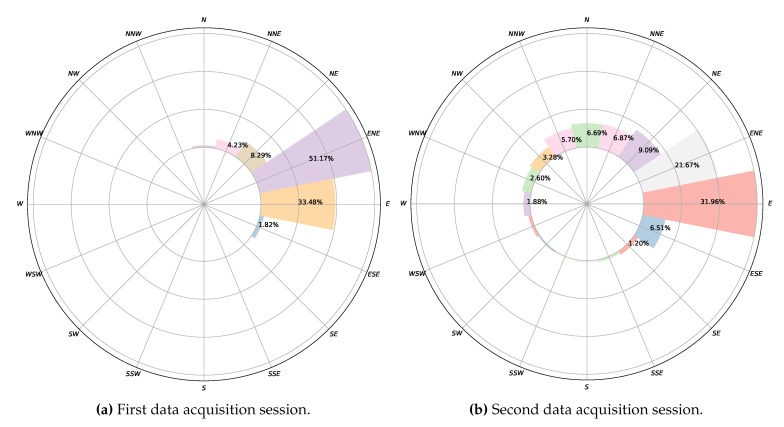
Wind direction distributions in both experimental sessions; in (**a**,**b**), values for wind directions with percentages less than 1% are not shown.

**Figure 17:**
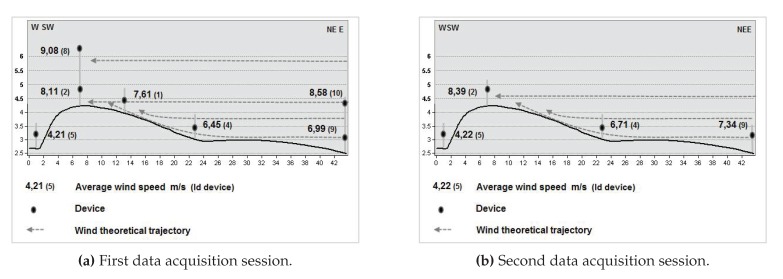
Topographic profile along the longitudinal axis of a tongue dune, with indication of the average speeds recorded by different sensors located on that axis for both data acquisition sessions.

With respect to the wind data collected during this experimental study, a total of 3220 data points were registered during the first data acquisition session, and 6560 during the second. With respect to wind directions, as can be seen in Figure 16a, in the first experiment, 84.65% of the wind measurements were from ENE and E. For the remaining 15.35% of the data, the wind blew from different directions. In the second experiment, as shown in Figure 16b, 69.23% of the wind components were from NE, ENE, E, and ESE, and 13.56% from N and NNE. For the rest of the data, 17.21%, the wind blew from several directions. Figure 13 and Figure 14 summarize the data acquired in each session, showing the average wind speeds and angles and the standard deviations for all the deployed sensors. The topographic profile of the longitudinal axis of the dune is shown for each experiment in Figure 17, as well as the arrangement of the wind sensors along that axis, which is of interest for the analysis of wind variation along it.

**Figure 18:**
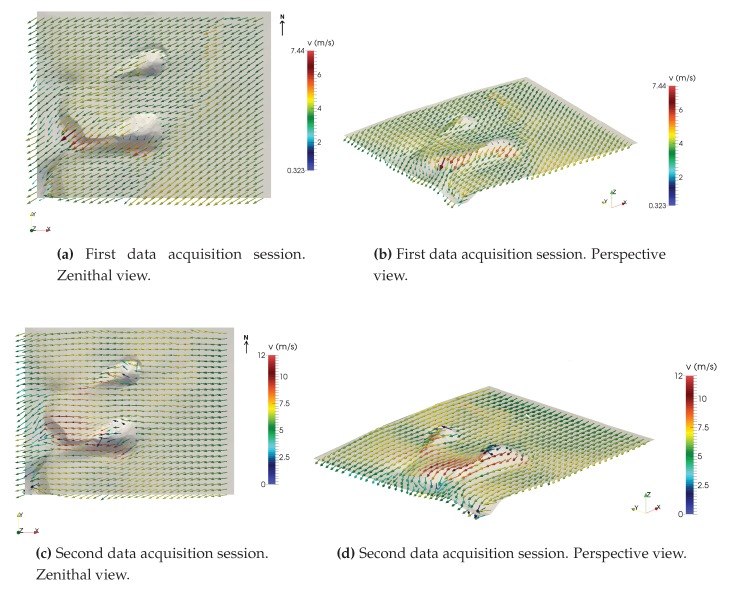
Wind model results for a given instant *t* with the collected data in both data acquisition sessions.

Figure 18a,b show the model’s output for a given instant t, based on wind data captured during the first data acquisition session. Similarly, Figure 18c,d correspond to the results produced by the model for a specific instant of time for the second session. The ability to build a model of the wind field on the terrain at any instant of time allows us to identify the shape of the dunes under study. In this case, our main interest is in studying the genesis of tongue dunes in this specific area, from the interference of two nebkhas formed by two species of *Traganum moquinii* in the aeolian sedimentary dynamics. According to [46], the dune studied should be in an advanced phase of its evolution, depicting a clear parabolic shape. In November 2014, it was advancing at an approximate speed of 7 m per month, which is the highest registered displacement rate [46]. The origin of this dune corresponds to the accumulation of sand in the backshore, windward of the vegetation specimens [46]. The shrub vegetation retains part of the sediment and modifies the surrounding wind conditions, concentrating the highest energy flux in the central part of the landform. In this way, the dune advances towards the inner dune field, transported by the effective NNE winds, changing its morphology until it becomes a tongue dune—the moment at which this study begins. From this time onward, the tongue dune begins to elongate, penetrating more and more inside the dune field. Finally, at distances longer than 10 m, the dune gets rid of the shrubs and their direct influence and advances, converted into a barchan dune.

From an environmental point of view, a model has been developed, fitted to experimental wind and altitude data that allows characterisation of changes in the direction and intensity of the wind and its interaction with a pair of nebkhas formed by the presence of *Traganum moquinii* specimens in an arid foredune. The consequence of this interaction is, as expected, a reduced wind speed around the plants and higher wind speeds leeward of the specimens and their associated dunes. This fact explains the formation of these tongue dunes as the result of the coalescence of nebkha tails. The highest wind speeds are observed at the brink of the tongue dune, where the air flux is compressed. Zones where the model is not in good agreement with the data are those located leeward of the vegetation. In particular, at the locations of stations 2, 3, and 7 (see Figure 11a and corresponding locations in Figure 18a,b), the model underestimates the wind speed in the first acquisition session. Taking into consideration the roughness length concept, the model states that from the selected roughness length upwards there is no object interfering in the aeolian flux. This circumstance is, clearly, unreal in the area occupied by the vegetation, as the shrubs may have a high density in their first 0.10 m and force a null wind speed. Given the characteristics of *Traganum moquinii* canopy, its density diminishes with height to approximately 0.60 m. Thus, as we ascend in height, two circumstances appear that are not taken into account in the wind model. One is that wind permeability increases, and the other is that a fraction of the wind energy is consumed by canopy motion and, consequently, wind intensity diminishes. In order to obtain a better agreement between the model and the observed data, a higher density of wind measuring devices would be needed leeward and at several different heights around the vegetation.

As to the DIY data acquisition system we have designed, developed and deployed for this experimental study, and considering the initial design goals described previously in Section 3, Section 3.1.1, a description is provided below of the extent to which each of those design goals has been met:

**Cost** Table 3 shows a summary of the approximate cost of each of the wind sensing devices which constitute the data acquisition system. Each item’s cost is approximate, as the value may differ slightly depending on the seller or provider. The total cost for a wind sensing device is well below the cost of a typical commercial 3D ultrasonic anemometer, sometimes by as much as one order of magnitude compared to research-grade wind sensors. In this case, eleven wind sensing devices were built and used for the experimental study. Evidently, high-priced ultrasonic anemometers are ideal for studies which require wind data acquisition at lesser spatial scales where wind turbulence plays a key role. However, this is not the case for the spatial scale of the experimental study we carried out. Consequently, the more cost-effective DIY approach considerably reduced the cost barrier of this experimental study. As to the costs of the base station, Table 3 also lists the cost of a base station comprised of a Raspberry Pi board and a mobile tablet device, which was the configuration used in both data acquisition sessions. Bearing in mind that a laptop running GNU/Linux can also act as a base station, as mentioned earlier in Section 3, in this case, the base station cost will amount to the cost of the laptop computer, the XBee Explorer USB interface and the XBee communication module.
**Flexible system parametrization** The embedded software developed for the wind sensing devices, along with the control and monitoring software developed for the base station, allowed the configuration of a variety of parameters which can affect a data acquisition session. It is possible to adjust the data sampling periods in the wind sensing devices and the log files in both kinds of nodes, the base station and the data acquisition devices. As to the calibration and homing procedures of the wind vane, the time window can be adjusted as well as the beep patterns. Other additional aspects which can also be altered include, for example, the temperature interval to indicate overheating situations through the temperature status indicator on the control and monitoring interface, the interface timeout which indicates that a wind sensing device has communication problems, or the period of the base station’s PPS broadcast.
**Autonomy** In relation to power autonomy, an experimentally determined autonomy of slightly more than 30 h of continuous operation was observed for each wind sensing device, which is well above the initial requirement of 10 h of power autonomy. Furthermore, as previously mentioned in Section 3, the current design permits, if required, the use of a solar panel to charge each wind sensing device’s battery pack. This would allow deployment of the devices for longer experimental sessions. Longer power autonomy is also feasible, if required, by taking advantage of some of the power-saving features present in Arduino UNO’s ATmega328P microcontroller, namely the use of its power-saving sleep modes, the possibility of deactivating some of the microcontroller’s on-chip peripherals when not used, and the option of dynamically changing its system clock [80].
**Temperature concerns** The control and monitoring interface of the base station allowed the monitoring of each device in case excessive temperatures were reached and actions needed to be taken. The highest temperatures that were recorded during the data acquisition sessions that we carried out were well below 50 ${}^{{}^{\circ}}$C, and so no intervention was required. It should be noted that, as a precautionary measure, the compartment containing the electronics of each wind sensing device, shown in Figure 3b, was protected in both experimental sessions with a white cloth cover, visible in Figure 12a,b, to mitigate any problem of overheating due to solar radiation.
**Easy deployment and use** All the wind sensing devices were battery powered and communicated wirelessly, facilitating their deployment as no cabling was required. Equally, as they are light and small devices, their on-site transportation can be carried out by one person on most occasions. As to the initial calibration and homing procedures for each device when deployed, these are relatively simple and can be carried out by people without any special training, with the only requirement being to bring a compass for the homing procedure. No special training is required either to use the control and monitoring interface of the base station. The team that conducted the experiments found the data acquisition system equipment easy to use on-site, and no extra support was required.
**Wireless communications** As mentioned above, the wireless communications system facilitated deployment as there was no need to deploy wired connections between the wind sensing devices during the on-site experimental sessions, which is usually logistically very cumbersome. In addition, as Figure 1 illustrates, the data acquisition system presents a *star* network topology, with the base station as the central node. In other words, the wind sensing devices do not communicate between themselves, but instead only on a P2P basis with the base station. This star network topology setting is valid for deployments in areas where all the wind sensing devices are within reach of the base station and can communicate individually with it. For studies in wider areas, covering larger areas, it is possible to configure the XBee PRO S1 802.15.4 modules, changing their firmware, with a communication protocol capable of dynamic rerouting and packet relaying which will allow deployment of the data acquisition system as a *mesh* topology, specifically using the protocol DigiMesh [81]. Using this configuration, as a mesh topology, deployment of the data acquisition system in more extensive areas is possible as the data can be routed to the base station from the furthest wind sensing devices thanks to packet retransmission between the XBee modules.
**Reach** The star topology used during the experimental sessions was within reach of the XBee PRO S1 802.15.4 modules, which, according to their specifications [50], can extend to approximately 1.6 km in favourable conditions. This range is well above that required for the study area of approximately 50 × 50 square meters where the data acquisition sessions were performed. There were no communication problems between the base station and the wind sensing devices during the sessions. In fact, thanks to the reach of XBE PRO S1 802.15.4 modules, the data acquisition system has already been deployed to study bigger areas, specifically in an experimental study covering an area of 27.76 ha, the results of which have been published in [82]. Moreover, as mentioned in the previous paragraph, if required, the data acquisition system can be configured using a mesh network topology, which will enable experimental deployments with greater reach and hence the coverage of more extensive areas.
**Reliability** During the data acquisition sessions which were carried out for this environmental study, the system was continuously monitored through the control and monitoring interface running in the base station. No faulty situation was detected during the sessions. For the length of the experimental sessions, the collected data were stored in both the base station’s secondary memory and in each wind sensing device’s micro-SD card. After the sessions had concluded, it was found that one of the wind sensing devices (device No. 10) was displaying incorrect behaviour in the course of the offline processing of the collected wind data in the second data acquisition session, and for this reason has been omitted in Figure 14. This erroneous behavior was not detected on-site. The hypothesis for the cause of this malfunction was a bad wire contact due to sand and humidity during the session.

Finally, in Table 4, a summary is provided of the main features of the DIY wireless wind data acquisition system we have developed and which was deployed in the experimental study described in this section.

### 4.4. Further Experimental Studies

In addition to the experimental study described above, another environmental analysis was performed in a different location within the same dune field and using the same DIY wireless data acquisition system. The purpose of this study was to analyse the influence of tourist facilities and buildings situated at the border limits of the dune field on the dynamics of the dune system, since they can modify the wind behaviour and have a significant impact on the area. The zone under analysis for this study was an area of 27.76 ha, with the wireless acquisition system deployed in several experimental sessions on 24–25 March 2017. In particular, five data acquisition sessions were carried out. For each session, ten wind sensing devices were deployed on five towers, each with a pair of devices at two different heights of 0.4 m and 2.1 m. Each experimental session covered one of the five transects into which the study area was split. The methodological approach used to deploy the system for each session was the same as described previously in this section, with the collected wind data integrated into a DEM of the terrain, obtained in this case using digital orthophotos from a photogrammetric drone flight on-site covering the whole area. In addition, the precision of the topographic data derived from the drone flight was tested using ground data collected on-site with a topographic total station. Further details can be found in [82].

## 5. Conclusions

The development of a low cost and wireless wind data acquisition system that allows multiple synchronized wind measurement points enabled the data-intensive modelling of the interaction of an arid foredune system with its shrub vegetation. Wind data acquisition in dune field environments is usually expensive due to the high cost of the instrumentation used in this type of environmental study. In fact, the experimental study and modelling described in this paper would not have been possible with expensive standard research-grade sensors. However, it was possible to considerably reduce this fundamental cost barrier with the DIY data acquisition system which we designed and developed. The system was experimentally applied to the study of the genesis and dynamics of tongue dunes in the arid dune field of Maspalomas (Gran Canaria, Canary Islands, Spain), a new finding which is of interest at a local level for the management of this dune field, and, at a general level, as knowledge that can be applied to other similar dune areas around the world. Given the scenario of global change, the monitoring of environmental aspects is very important in coastal areas as many of them present aeolian sedimentary environments in arid zones which need special protection. A more cost-effective approach to such environmental studies is of general interest, but is especially true for developing countries with less research funding [83]. In a subsequent experimental work [82], not described in this manuscript, the data acquisition system was again successfully deployed covering a larger extension. On this occasion, it was used to study the influence of tourist constructions on the limits of the same dune field as they significantly affect wind behavior and, consequently, sand transportation and dune formation.

## Figures and Tables

**Table 1:** Data comparison. Error of measurements.

Wind Data Error Comparison	Regression Analysis Coeficient $R^{2}$	Mean Bias Error (MBE)	Root Mean Square Error (RMSE)
**Speed**	0.950862	−0.167 m/s	0.468 m/s
**Direction angle**	0.871838	1.107${}^{{}^{\circ}}$	3.557${}^{{}^{\circ}}$

**Table 2:** Data acquisition sessions. Location and elevation of sensor nodes.

First Data Acquisition Session					Second Data Acquisition Session				
**Node**	**X (UTM)**	**Y (UTM)**	**Elevation (m)**	**Level above Ground (m)**	**Node**	**X (UTM)**	**Y (UTM)**	**Elevation (m)**	**Level above Ground (m)**
1	443871.613	3069222.632	4.45	0.50	1	443860.808	3069217.213	3.26	0.40
2	443865.393	3069220.715	4.86	0.50	2	443865.377	3069220.706	4.81	0.45
3	443868.921	3069225.821	5.83	1.90	3	443862.589	3069226.298	3.97	1.56
4	443881.383	3069224.574	3.47	0.50	4	443881.386	3069224.595	3.47	0.50
5	443860.019	3069220.822	3.17	0.50	5	443859.917	3069220.810	3.17	0.50
6	443868.921	3069225.821	4.43	0.50	6	443862.589	3069226.298	2.78	0.37
7	443866.896	3069215.118	5.67	0.50	7	443866.874	3069215.133	5.67	0.50
8	443865.393	3069220.715	6.26	1.90	8	443860.808	3069217.213	4.75	1.90
9	443901.172	3069228.772	3.05	0.58	9	443901.172	3069228.772	3.05	0.58
10	443901.172	3069228.772	4.37	1.90	10	443868.054	3069227.449	4.14	0.50
11	443866.896	3069215.118	7.07	1.90	11	443864.411	3069213.071	4.27	0.41

**Table 3:** Cost of components.

Wireless Wind Sensing Device		Base Station	
Arduino UNO rev. 3 μ-controller	≈ 23.00 €	Raspberry Pi 3 B+	≈ 38.00 €
Wireless SD shield	≈ 23.00 €	XBee Explore USB	≈ 22.00 €
XBee PRO S1 802.15.4 60 mW	≈ 33.00 €	XBee PRO S1 802.15.4 60 mW	≈ 33.00 €
LiPo Rider Pro	≈ 13.50 €	Mobile Tablet 10"	≈ 250.00 €
LiPo 3-battery Pack 6000 mAh /3.7v	≈ 38.00 €	**Total**	**≈ 343.00 €**
TMP36 temperature sensor	≈ 2.00 €		
Buzzer/Beeper	≈ 4.00 €		
US Digital MA3 based wind vane	≈ 50.00 €		
**Total**	**≈ 186.50 €**		

**Table 4:** System summary.

System Features		Wind Sensing Device Features	
**Wireless technology**	XBee 802.15.4/DigiMesh	**Embedded system**	Arduino UNO rev. 3 based
**System components**	a base station and multiple wireless wind sensing devices	**On board sensors**	anemometer, wind vane and temperature sensor
**Data store**	locally in sensing devices’ micro-SD cards and globally in base station’s secondary memory	**Anemometer error estimation**	0.468 m/s
**Data time synchronization**	based on base station’s clock or GPS based.	**Wind vane error estimation**	3.557${}^{{}^{\circ}}$
**Range**	in *star* topology: area of maximum radius of 1.6 km. in *mesh* topology: potentially unlimited areas using DigiMesh’s rerouting and packet relaying.	**Autonomy**	with fully charged batteries: about 30 h solar-powered: potentially unlimited
**Costs**	wind sensing device: approx. 186.50 € per unit base station: approx. 343.00 €		
